# Long-term cardiovascular inflammation and fibrosis in a murine model of vasculitis induced by *Lactobacillus casei* cell wall extract

**DOI:** 10.3389/fimmu.2024.1411979

**Published:** 2024-06-25

**Authors:** Ana Paula Lombardi Pereira, Emily Aubuchon, Debbie P. Moreira, Malcolm Lane, Thacyana T. Carvalho, Thassio R. R. Mesquita, Youngho Lee, Timothy R. Crother, Rebecca A. Porritt, Waldiceu A. Verri, Magali Noval Rivas, Moshe Arditi

**Affiliations:** ^1^ Laboratory of Pain, Inflammation, Neuropathy, and Cancer, Department of Pathology, Londrina State University, Londrina, Brazil; ^2^ Department of Pediatrics, Division of Infectious Diseases and Immunology, Guerin Children’s at Cedars-Sinai Medical Center, Los Angeles, CA, United States; ^3^ Infectious and Immunologic Diseases Research Center (IIDRC), Department of Biomedical Sciences, Cedars-Sinai Medical Center, Los Angeles, CA, United States; ^4^ Smidt Heart Institute, Cedars-Sinai Medical Center, Los Angeles, CA, United States; ^5^ Sanford Burnham Prebys Medical Discovery Institute, La Jolla, CA, United States

**Keywords:** abdominal aorta dilations, aortitis, coronary artery aneurysms, fibrosis, Kawasaki disease, long-term inflammation, vasculitis.

## Abstract

**Background:**

Kawasaki disease (KD), an acute febrile illness and systemic vasculitis, is the leading cause of acquired heart disease in children in industrialized countries. KD leads to the development of coronary artery aneurysms (CAA) in affected children, which may persist for months and even years after the acute phase of the disease. There is an unmet need to characterize the immune and pathological mechanisms of the long-term complications of KD.

**Methods:**

We examined cardiovascular complications in the *Lactobacillus casei* cell wall extract (LCWE) mouse model of KD-like vasculitis over 4 months. The long-term immune, pathological, and functional changes occurring in cardiovascular lesions were characterized by histological examination, flow cytometric analysis, immunofluorescent staining of cardiovascular tissues, and transthoracic echocardiogram.

**Results:**

CAA and abdominal aorta dilations were detected up to 16 weeks following LCWE injection and initiation of acute vasculitis. We observed alterations in the composition of circulating immune cell profiles, such as increased monocyte frequencies in the acute phase of the disease and higher counts of neutrophils. We determined a positive correlation between circulating neutrophil and inflammatory monocyte counts and the severity of cardiovascular lesions early after LCWE injection. LCWE-induced KD-like vasculitis was associated with myocarditis and myocardial dysfunction, characterized by diminished ejection fraction and left ventricular remodeling, which worsened over time. We observed extensive fibrosis within the inflamed cardiac tissue early in the disease and myocardial fibrosis in later stages.

**Conclusion:**

Our findings indicate that increased circulating neutrophil counts in the acute phase are a reliable predictor of cardiovascular inflammation severity in LCWE-injected mice. Furthermore, long-term cardiac complications stemming from inflammatory cell infiltrations in the aortic root and coronary arteries, myocardial dysfunction, and myocardial fibrosis persist over long periods and are still detected up to 16 weeks after LCWE injection.

## Introduction

Kawasaki disease (KD) is a febrile systemic vasculitis that mainly affects children under the age of 5, leading to inflammation in the walls of blood vessels, particularly the coronary arteries (CA) (1, 2). The syndrome is characterized by a high fever that persists for at least five days and the appearance of several clinical manifestations, such as changes in the oral mucosa and cracking/fissuring lips, conjunctivitis, skin rash, edema and desquamation of the extremities (hands and feet), and cervical lymphadenopathy (1, 2). Despite significant efforts over the past few decades to determine the cause of KD, its etiology remains unknown. It is hypothesized that an infectious agent(s) causes KD, possibly a virus, which triggers an inflammatory response targeting cardiovascular tissues (3, 4). However, no specific infectious agent has consistently been associated with KD (5).

If left untreated, KD vasculitis can result in serious cardiac complications, and indeed, KD is the most prevalent acquired heart disease in children in developed countries (1, 2, 6). CA lesions (CALs) are the most prevalent manifestation and may eventually lead to acute myocardial infarction, which in some rare cases can be fatal (7–10). CALs usually develop during the acute phase of KD (febrile phase), which lasts up to 10 days after fever onset (1). While intravenous immunoglobulin (IVIG) treatment effectively reduces the uncontrolled inflammatory response, around 4–5% of IVIG-treated KD patients still develop CALs (1, 11, 12). Moreover, approximately 10% to 20% of children with KD do not respond to IVIG and are at even higher risk of developing severe cardiovascular complications (1, 13, 14). CALs can persist and progress for months and even years following the initial diagnosis, causing long-term cardiac complications that can extend into adolescence and adulthood (15–18).

Orenstein et al. reported that KD vasculopathy develops in three linked pathological processes involving tissue infiltration of innate and adaptive immune cells (19, 20). The first process, necrotizing arteritis (NA), starts at the endothelial layers of the CA. It is followed by subacute/chronic (SA/C) vasculitis and finally by luminal myofibroblast proliferation (LMP) (19, 20). However, other pathological reports on autopsy findings have not observed this SA/C persistent vessel inflammation (21–23).The NA stage consists of massive infiltration of polymorphonuclear cells, especially neutrophils, which secrete inflammatory mediators including cytokines, matrix metalloproteinases, elastase, and other enzymes (19, 20). These mediators destroy the elastic layers and media, progressively causing the structural support of the CA to break down and the subsequent development of aneurysms and dilations (19, 20). NA is self-limited, lasting for two weeks, followed by granulation tissue and scar formation that occurs months or years after the acute phase of KD (19, 22, 23). This chronic phase is an asynchronous process involving infiltration within the CA tissue of T lymphocytes, especially CD8^+^ T cells, IgA plasma cells, and scattered macrophages (19, 21, 24–27). The granulation stage leads to LMP and might be observed for months to years after disease onset (19). During LMP, myofibroblasts proliferate in the adventitia layer and extend the lesion towards the lumen. Ultimately, persistent granulation and scarring processes result in complete arterial wall destruction and the formation of coronary artery aneurysms (CAA) that can cause CA stenosis, leading to myocardial ischemia, ischemic heart disease, thrombosis, or rupture (19).

Detailed histological studies of cardiac tissue from KD patients have shown critical morphological and histological alterations years after initial diagnosis (9, 19). Fatal outcomes related to KD were previously believed to occur only within 60 days of disease onset and, therefore, were exclusively associated with acute and subacute phases (1). However, more recent studies have reported fatal cases due to cardiac complications, with ischemic heart diseases being the most frequent, with an increased likelihood of occurrence during the first year after KD diagnosis and even years after that (9, 15–17, 28–30). Moreover, a potential link between myocardial infarction deaths and undiagnosed cases of KD has been suggested (15–18).

Given the possible long-term complications of KD (15, 31), investigating potential cardiovascular involvement beyond the acute phase of KD and long-term immunopathogenesis and histological alterations is warranted. However, such studies are challenging due to not only the complexity of the disease but also the limited access to human heart tissue samples. As an alternative to patient samples, the *Lactobacillus casei* wall extract (LCWE)-induced mouse model of KD-like vasculitis is a well-described and widely accepted animal model (5, 32). This model reproduces the key pathological features of KD, including coronary arteritis, aortitis, myocarditis, abdominal aorta aneurysms, myocardial dysfunction with mildly diminished ejection fraction (EF), and even electrocardiogram (EKG) changes and arrhythmias that may occur in clinical KD (5, 33–38). Furthermore, it has also been successfully used to translate treatment responses to IVIG, anti-tumor necrosis factor (TNF)-α, and IL-1R antagonist (IL-1Ra; Anakinra) (35, 36, 39, 40). Acute myocarditis and chronic scarring of the CA with the formation of stenotic CA fragments are also observed in the LCWE-induced KD model (5, 38), similar to the fibrotic lesions that might lead to the development of long-term cardiac complications in children with KD (15, 18). Here, we used the LCWE-induced KD-like mouse model to investigate the long-term immunological and pathological changes of LCWE-induced cardiovascular inflammation and cardiac dysfunction over an extended period of the disease.

## Results

### LCWE induces long-term cardiovascular lesions

**Figure 1:**
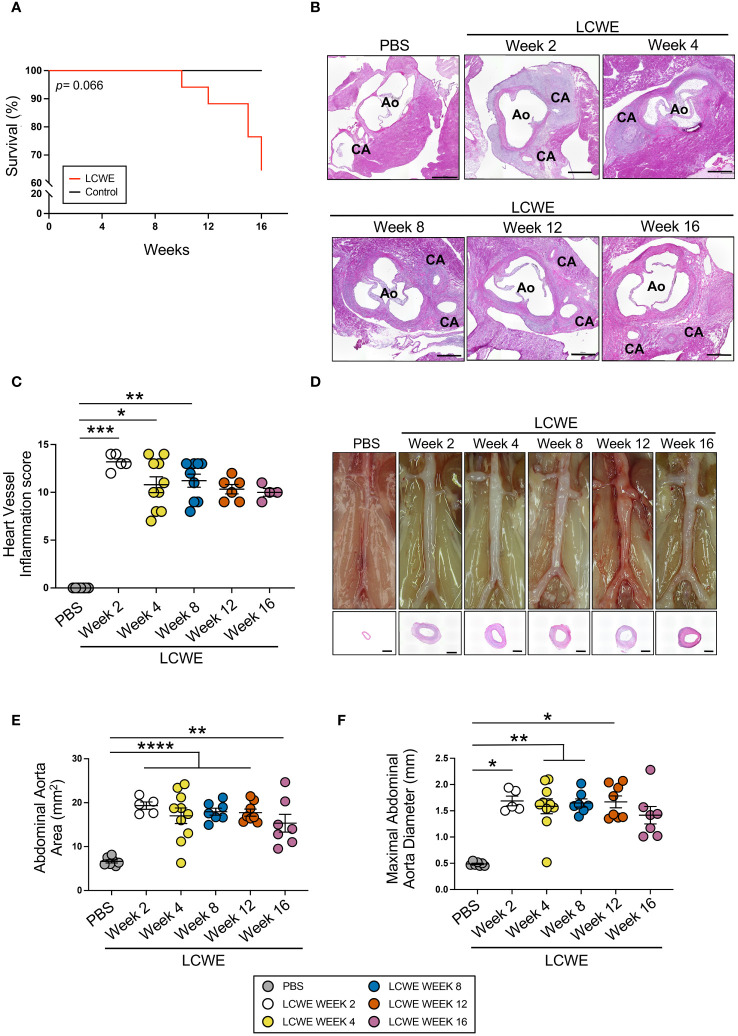
Long-term progression of LCWE-induced KD-like vasculitis. **(A)** Survival curves of PBS and LCWE-injected mice (n= 8–17 mice/group). **(B)** Representative H&E-stained heart tissue of PBS or LCWE-injected mice at weeks 2, 4, 8, 12, and 16 post-LCWE-injection. Scale bars: 500 μm. **(C)** Heart vessel inflammation scores of PBS and LCWE-injected mice at 2, 4, 8, 12, and 16 weeks post-LCWE injection (n= 4–10/group). **(D)** Representative pictures of the abdominal aorta area and H&E staining of the abdominal aorta cross-sections. Scale bars: 500 μm. **(E, F)** Abdominal aorta area **(E)** and maximal abdominal aorta diameter **(F)** measurements of PBS and LCWE-injected mice at the indicated time points post-LCWE injection (n= 7–9/group). Survival analysis was done by Log-rank test (Mantel-Cox) **(A)**. Data are presented as mean ± SEM. * p<0.05, **p<0.01, ***p<0.001, ****p<0.0001 by one-way ANOVA with Tukey post-test **(E)** or Kruskal-Wallis with Dunn’s post-test **(C, F)**. CA indicates coronary artery; and Ao, aorta.

We first investigated the long-term pathology and persistence of LCWE-induced KD-like vasculitis by injecting WT mice with either PBS or LCWE and assessing heart vessel inflammation and abdominal aorta dilation 2, 4, 8, 12, and 16 weeks later (**Figure 1**). We observed a trend of increased mortality in LCWE-injected mice compared with control PBS-injected mice, resulting in 35% mortality at 16 weeks post-LCWE injection (**Figure 1A**). In heart tissues of LCWE-injected mice, we observed extensive infiltrations of inflammatory cells in the area surrounding the CA and several cases of complete stenosis of the CA, even at the earliest time point (**Figure 1B**, **Supplementary Figures S1A, B**). Indeed, at two weeks post-LCWE injection, 50% of LCWE-injected mice exhibited vascular lesions resulting in complete occlusion of the affected CA (**Supplementary Figures S1A, B**). Compared with PBS control mice, LCWE-injected mice developed severe heart inflammation, as demonstrated by a higher heart vessel inflammation score (aortitis and coronary arteritis), starting at 1 week (data not shown) which reached its maximal level at week 2 post-LCWE injection and remained elevated at weeks 4, 8, 12 and 16 post-LCWE (**Figure 1C**). LCWE-injected mice also exhibited abdominal aorta dilatations and aneurysms, which were localized exclusively in the infrarenal part of the abdominal aorta, as measured by abdominal aorta area as well as maximal abdominal aorta diameter (**Figures 1D–F**). Hematoxylin and eosin (H&E)-stained cross-sections of the abdominal aorta indicated intimal infiltration and increased aortic wall thickness (**Figure 1D**). However, the severity of cardiovascular lesions reached a plateau, and heart vessel inflammation and the size of abdominal aorta dilations were similar in LCWE-injected mice from weeks 2–4 to week 16 (**Figures 1C, E, F**). These results indicate that LCWE-induced coronary arteritis and abdominal aorta dilations do not regress and persist beyond the maximal acute injury phase of the disease.

### Systemic alterations in immune cell composition during the acute and long-term phases of LCWE-induced KD-like vasculitis

**Figure 2:**
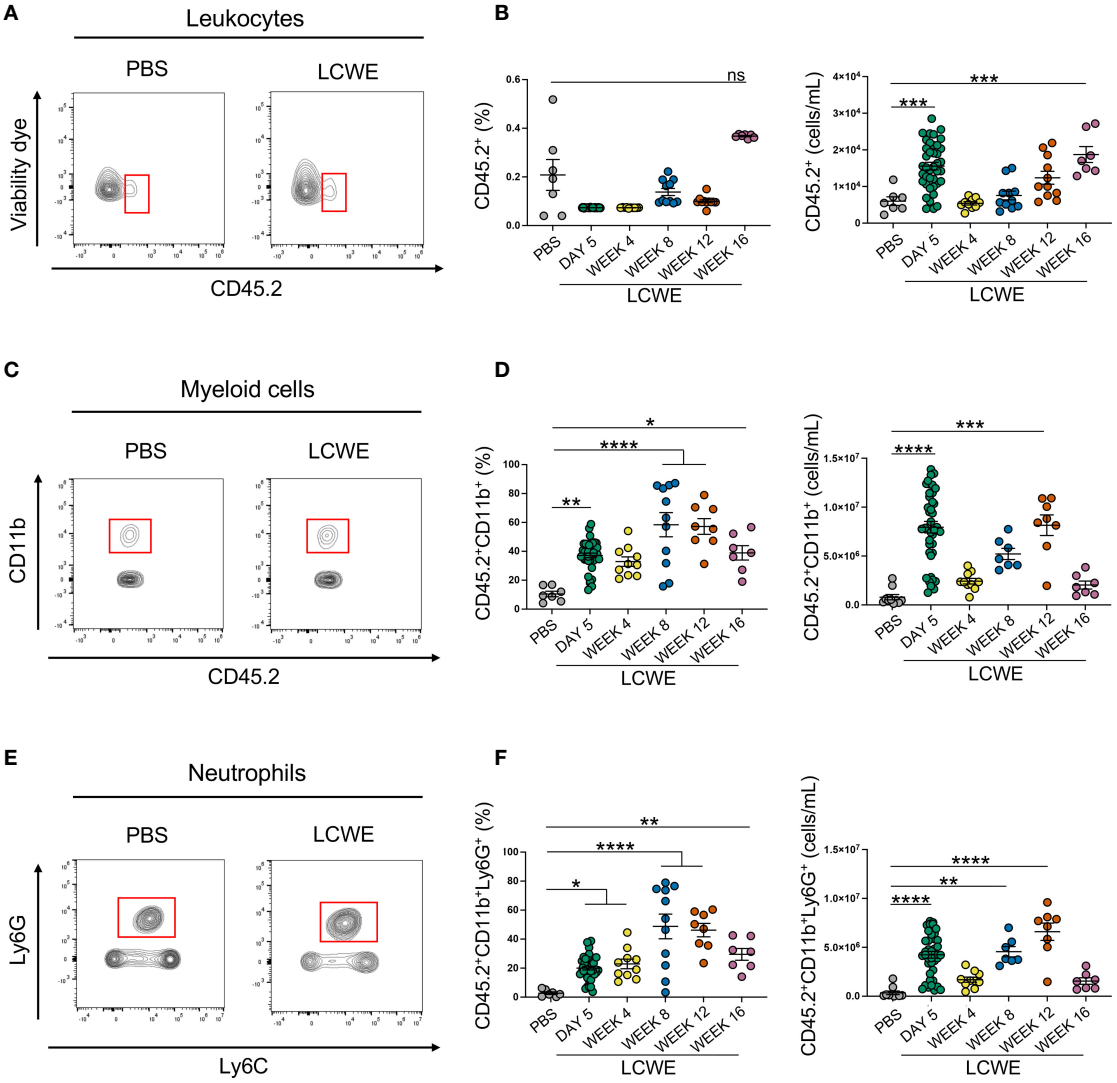
Increased circulating frequencies of immune cells during LCWE-induced KD-like vasculitis. **(A, B)** Flow cytometry plots **(A)**, frequencies, and cell numbers **(B)** of live CD45.2^+^ cells in the blood of PBS and LCWE-injected mice. **(C, D)** Flow cytometry plots **(C)**, frequencies, and cell numbers **(D)** of myeloid cells (live CD45.2^+^ CD11b^+^) in the blood of PBS and LCWE-injected mice. **(E, F)** Flow cytometry plots **(E)**, frequencies, and cell numbers **(F)** of neutrophils (live CD45.2^+^ CD11b^+^ Ly6G^+^) in the blood of PBS and LCWE-injected mice. Data are presented as mean ± SEM. and combined from 3 independent experiments (n=7–40/group). *p<0.05, **p<0.01, ***p<0.001, **** p<0.0001 by one-way ANOVA with Tukey post-test or Kruskal-Wallis test with Dunn’s post-test for nonparametric data. ns = not significant.

**Figure 3:**
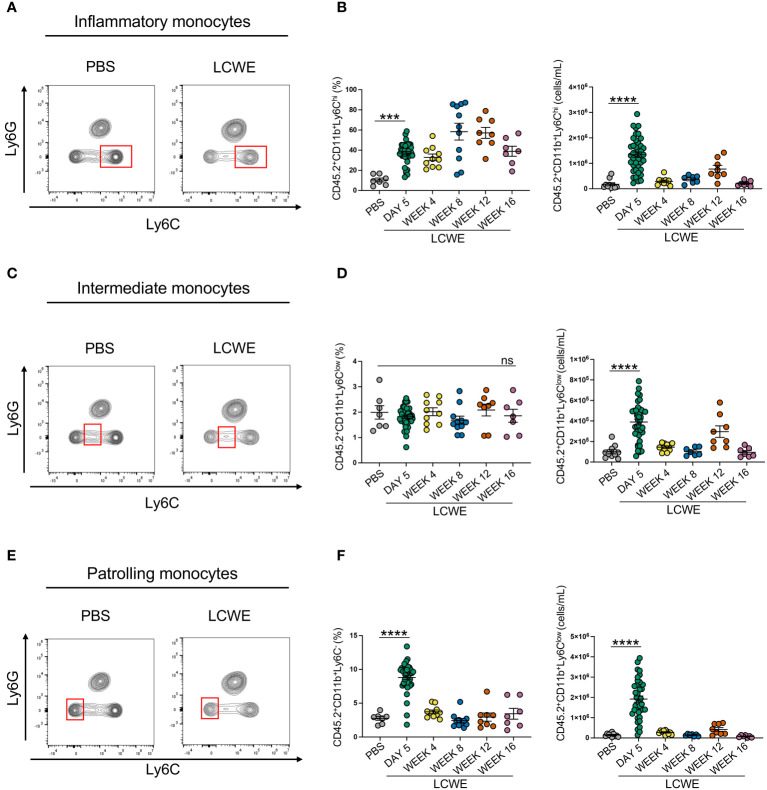
Increased circulating frequencies of monocytes during acute LCWE-induced KD-like vasculitis. **(A, B)** Flow cytometry plots **(A)**, frequencies and cell numbers **(B)** of inflammatory monocytes (live CD45.2^+^ CD11b^+^ Ly6C^high^) in the blood of PBS and LCWE-injected mice (n=7–10 per group). **(C, D)** Flow cytometry plots **(A)**, frequencies and cell numbers **(B)** of intermediate monocytes (live CD45.2^+^ CD11b^+^ Ly6C^low^) in the blood of PBS and LCWE-injected mice (n=7–10 per group). **(E, F)** Flow cytometry plots **(A)**, frequencies and cell numbers **(B)** of patrolling monocytes (live CD45.2^+^ CD11b^+^ Ly6C^-^) in the blood of PBS and LCWE-injected mice (n=7–10 per group). Data are presented as mean ± SEM. ***p<0.001, ****p<0.0001 by one-way ANOVA with Tukey post-test or Kruskal-Wallis test with Dunn’s post-test for nonparametric data. ns = not significant.

We next sought to determine the composition of circulating immune cells during the acute and long-term phases of LCWE-induced KD-like vasculitis. Peripheral blood was collected from PBS-injected control mice and LCWE-injected mice on day 5, week 4, 8, 12, and 16 after LCWE injection. Samples were analyzed by flow cytometry using specific markers for leukocytes (live CD45.2^+^ cells), myeloid cells (live CD45.2^+^ CD11b^+^ cells), neutrophils (live CD45.2^+^ CD11b^+^ Ly6C^low^ Ly6G^+^ cells), inflammatory monocytes (live CD45.2^+^ CD11b^+^ Ly6G^–^ Ly6C^high^ cells), intermediate monocytes (live CD45.2^+^ CD11b^+^ Ly6G^–^ Ly6C^low^ cells) and patrolling monocytes (live CD45.2^+^ CD11b^+^ Ly6G^–^ Ly6C^–^ cells) (**Figures 2A–F**, **Supplementary Figures S2A–D**). LCWE injection led to increased numbers of circulating leukocytes at day 5, with a second peak at week 16 (**Figures 2A, B**). The frequency and number of myeloid cells significantly increased at day 5 and week 12 post-LCWE injection compared to PBS-injected control mice (**Figures 2C, D**). Similarly, the frequency and number of circulating neutrophils increased at day 5 post-LCWE injection, peaked at weeks 8 and 12 post-LCWE injection, and progressively declined to reach their minimum at 16 weeks post-LCWE (**Figures 2E, F**). The different populations of monocytes were gated from CD45.2^+^CD11b^+^ Ly6C^+^ cells and classified according to their Ly6C expression (**Supplementary Figures S2A–D**). Inflammatory monocytes were selected based on high expression of Ly6C (Ly6C^high^), intermediate monocytes identified by low Ly6C^+^ (Ly6C^int^) expression, and patrolling monocytes identified based on negative expression of Ly6C (Ly6C^-^) (**Figures 3A–F**, **Supplementary Figure S2D**). The frequencies and numbers of the different circulating monocyte subsets were increased during the acute phase of LCWE-induced KD-like vasculitis at day 5 post-LCWE injection, but these differences did not persist to the later time points (**Figures 3A–F**). While the frequencies and numbers of inflammatory and patrolling monocytes peaked at day 5, only cell numbers of intermediate monocytes peaked at day five post-LCWE (**Figures 3A–F**). These results suggest that changes in systemic immune cell composition, particularly in frequencies and numbers of circulating neutrophils and myeloid cells, persist during the long-term phase of LCWE-induced KD-like vasculitis.

### Increased circulating levels of neutrophils are associated with the severity of LCWE-induced abdominal aorta dilations and cardiovascular lesions

**Figure 4:**
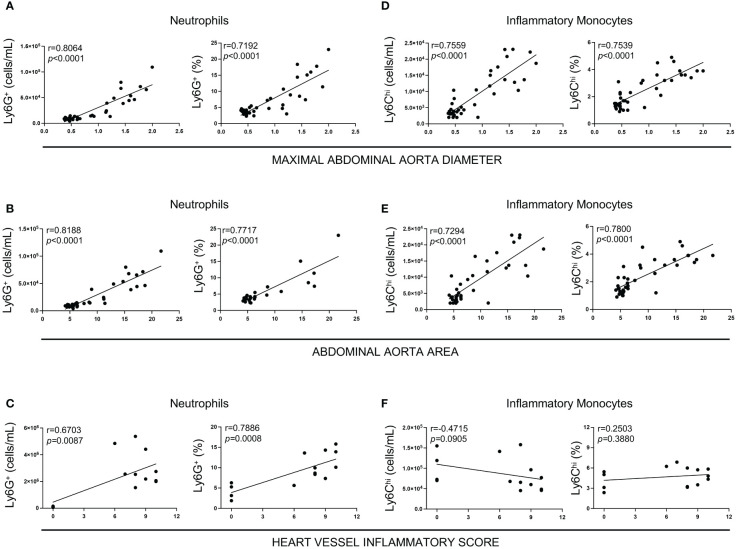
Correlation between heart vessel inflammation and abdominal aorta lesions with neutrophil and inflammatory monocyte levels in the peripheral blood 5 days after LCWE injection. **(A)** Spearman Correlation between neutrophils count and frequencies and maximum abdominal aorta diameter at two weeks post-LCWE. **(B)** Spearman Correlation between neutrophil counts and frequencies and abdominal aorta area at two weeks post-LCWE. **(C)** Spearman Correlation between neutrophilcounts and frequencies and heart vessels inflammatory score at two weeks post-LCWE. **(D)** Spearman Correlation between inflammatory monocyte counts and frequencies and maximum abdominal aorta diameter at 2 weeks post-LCWE. **(E)** Spearman Correlation between inflammatory monocytes count and frequencies and abdominal aorta area at 2 weeks post-LCWE. **(F)** Spearman Correlation between inflammatory monocyte counts and frequencies and heart vessels inflammatory score at two weeks post-LCWE. (n= 4–16 per group). R = correlation coefficient.

We next investigated if increased systemic frequencies and numbers of neutrophils observed at day 5 post-LCWE injection correlate with the severity of LCWE-induced cardiovascular lesions. Mice were injected with LCWE, blood was collected on day 5 post-LCWE injection, and circulating neutrophil frequencies and counts were determined by flow cytometric analysis. Heart vessel inflammation and maximal abdominal aorta diameter and area were assessed 14 days post-LCWE injection. Higher neutrophil frequencies and counts at day 5 strongly positively correlated with enlarged abdominal aorta dilations (**Figures 4A, B**) and the severity of heart vessel inflammation at day 14 post-LCWE (**Figure 4C**). Similarly, higher frequencies and counts of circulating inflammatory monocytes at day 5 post-LCWE positively correlated with the development of more severe abdominal aorta dilations at day 14 post-LCWE (**Figures 4D, E**). However, we did not find any significant correlation between the frequency of inflammatory monocytes at day 5 post-LCWE and the development of heart vessel inflammation at day 14 post-LCWE (**Figure 4F**). These data suggest that circulating neutrophil and inflammatory monocyte frequencies and counts are biomarkers indicative of disease severity and the development of LCWE-induced cardiovascular lesions in this experimental model of KD.

### Neutrophils and T cells infiltrate the inflamed coronary arteries of LCWE-injected mice

**Figure 5:**
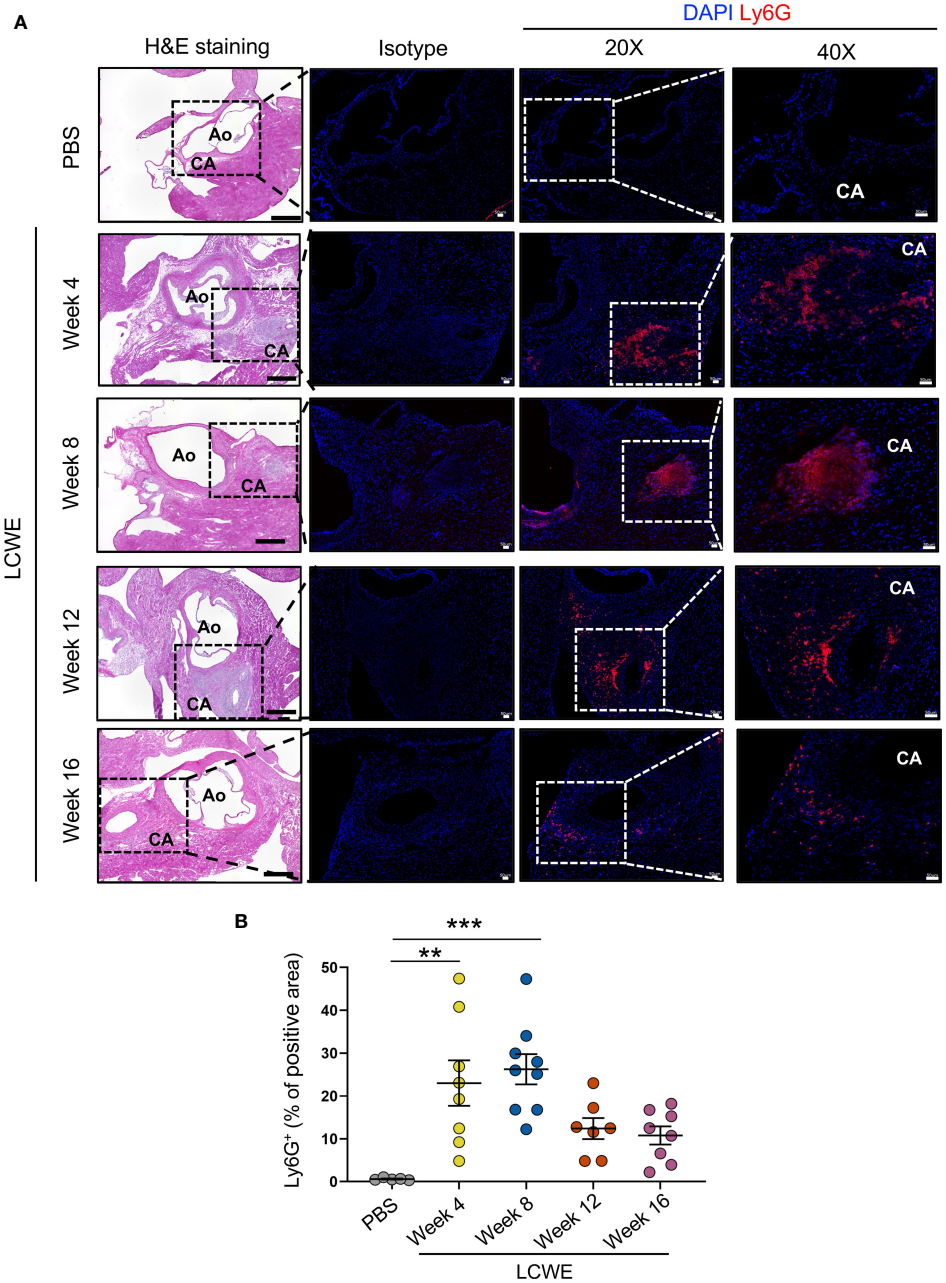
Neutrophilic infiltrations in heart tissues of LCWE-injected mice. **(A)** Representative H&E images and Ly6G immunofluorescent (IF) staining in heart tissues sections from PBS and LCWE-injected mice at different time points post-LCWE injection (n= 5–9 per group). DAPI (blue) was used to identify cell nuclei. **(B)** Quantification of Ly6G positive area in heart tissue from PBS and LCWE-injected mice at different time points post-LCWE injection (n= 5–9 per group). The result is expressed in the percentage of heart tissue positive for the Ly6G marker. Scale bar in H&E images: 500 μm. Scale bar in isotype, 20X, and 40X images: 50 μm. Data are presented as mean ± SEM. **p<0.01, ***p<0.001 by one-way ANOVA with Tukey post-test. CA, coronary artery; Ao, aorta.

Since the frequencies and numbers of circulating neutrophils increase in the acute phase of LCWE-induced KD-like vasculitis, and strongly correlate with the development of more severe cardiovascular lesions at day 14 post-LCWE (**Figures 2**, **4**), we next determined the presence of neutrophils in cardiac tissues by immunofluorescence (IF) staining. IF staining for Ly6G was performed on heart tissues from LCWE-injected mice at weeks 4, 8, 12, and 16 post-LCWE-injection, and the presence of neutrophils was quantified (**Figure 5**). Neutrophils in heart tissues of LCWE-injected mice also peaked at week 8 post-LCWE injection, progressively declined over time, and remained low at 16 weeks post-LCWE (**Figures 5A, B**). Neutrophils concentrated mainly around the inflamed CAs, and no neutrophils were detected in heart tissues from PBS-injected control mice (**Figures 5A, B**). Inflammatory T cells (CD3^+^) also infiltrate the CA of LCWE-injected mice and could be detected up to 16 weeks post-LCWE injection (**Supplementary Figure S3**). Our results indicate neutrophils contribute to the acute phase of the disease and decline overtime in the healing stages of the vasculitis.

### Fibrotic area in the coronary artery and aortic wall

**Figure 6:**
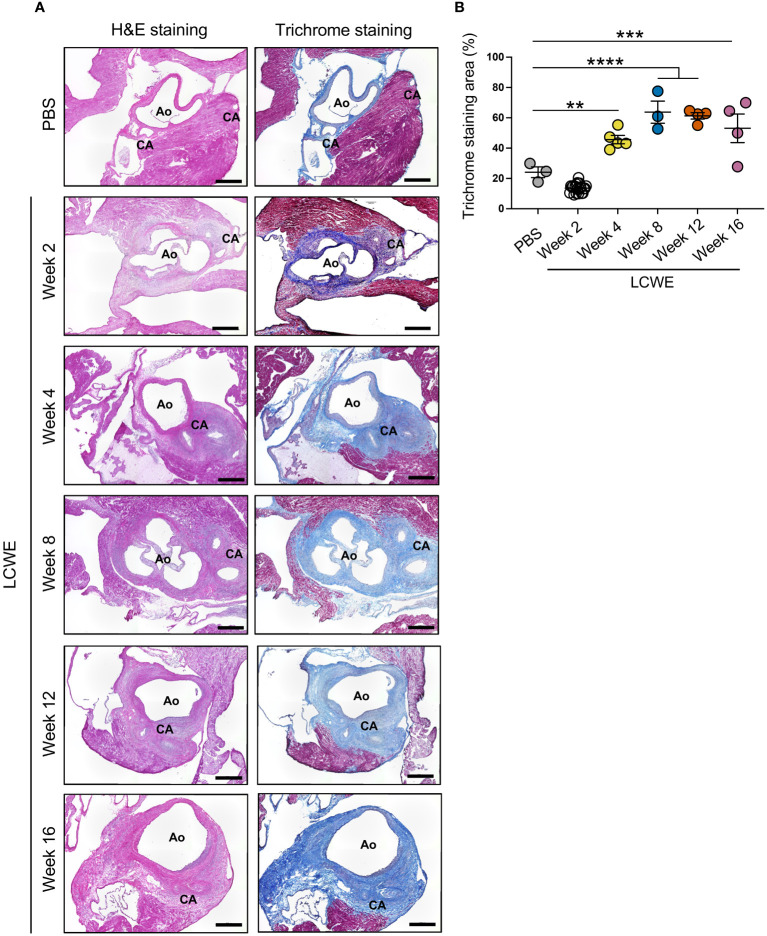
Long-term fibrosis in heart tissues from LCWE-injected mice. **(A)** Representative H&E staining and Masson’s trichrome staining of heart tissue sections from PBS control and LCWE-injected mice at different time points post-LCWE injection. **(B)** Quantification of the fibrotic area (blue) in heart tissues from PBS and LCWE-injected mice at 2, 4, 8, 12, and 16 weeks post-LCWE. Scale bars: 500 μm. Data are presented as mean ± SEM. **p<0.01, ***p<0.001 and ****p<0.0001, by one-way ANOVA with Tukey post-test (n=3–5 per group). CA, coronary artery; Ao, aorta.

We next sought to determine if LCWE-induced KD-like vasculitis leads to the development of persistent fibrosis near the CA, which represents LMP formation. Heart tissue sections from PBS control and LCWE-injected mice at different time points post-LCWE-injection were analyzed by Masson’s trichrome staining, and the fibrotic area was measured (**Figures 6A, B**). The deposition of collagen fibers was detected around the inflamed CA and aortic wall, in the same area where inflammatory cell infiltrations are observed (**Figures 6A, B**). Control mice injected with PBS showed minimum collagen staining, mainly restricted to the CA and aortic wall (**Figures 6A, B**). Heart tissue fibrosis around the CA, as determined by the percentage of trichrome-positive area, significantly increased starting from week 4 post-LCWE injection and remained elevated at weeks 12 and 16 post-LCWE (**Figures 6A, B**). We also observed diffuse interstitial fibrosis in the myocardium of mice 8 to 12 weeks after LCWE injection (**Supplementary Figure S4A**). These data suggest that LCWE-induced KD-like vasculitis leads to fibrosis around the CA with LMP, as well as myocardial fibrosis, which persists for up to 16 weeks post-LCWE injection.

### Myocarditis, long-term impairment of myocardial function, decreased ejection fraction, and left ventricle remodeling in LCWE-induced KD-like vasculitis

**Figure 7:**
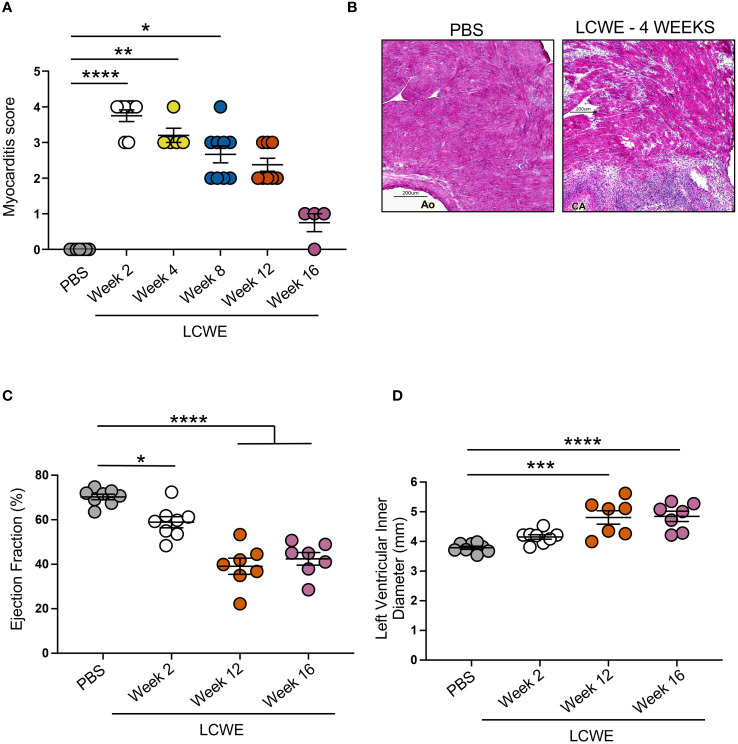
Myocarditis, reduced cardiac function and left ventricle remodeling in LCWE-injected mice. **(A)** Myocardial inflammation (myocarditis) score measured on H&E staining of heart tissue sections from PBS control and LCWE-injected mice at different time points post-injection. **(B)** Representative H&E staining of heart tissue sections from PBS and LCWE-injected mice at 4 weeks post-injection, showing myocardial inflammation and bridging fibrosis. Scale bar 200 μm. **(C)** Ejection fraction (EF) measured by transthoracic echocardiograph in control PBS and LCWE-injected mice at 2, 12, and 16 weeks pst-LCWE injection (n=7–8 per group). **(D)**. Left ventricle inner diameter measured by transthoracic echocardiograph in control PBS and LCWE-injected mice at 2, 12, and 16 weeks post-LCWE injection (n=7–8 per group). Data are presented as mean ± SEM. *p<0.05, **p<0.01, ***p<0.001, ****p<0.0001, by one-way ANOVA with Kruskal-Wallis post-test (n=4–9 per group) **(A)** or Tukey post-test (n=7–8 per group) **(C, D)**. CA, coronary artery; Ao, aorta.

Next, we aimed to evaluate whether myocardial functions, known to be affected by the intense heart inflammation (37–39), myocarditis, and myocardial fibrosis induced by LCWE injection in this murine model of KD-like vasculitis persist long-term. Myocardial inflammation score was assessed on H&E-stained heart tissue sections from PBS control and LCWE-injected mice at different time points of the disease, and the ejection fraction (EF) and the left ventricle (LV) internal diameter end-systole (LVIDd) parameters were analyzed by transthoracic echocardiogram. Compared with PBS-injected control mice, LCWE-injected mice showed significantly increased myocardial inflammation (myocarditis) scores starting at week 2 and continuing until week 12 post-LCWE injection (**Figures 7A, B**). Myocarditis mainly affected the areas adjacent to sites of aortitis and coronary arteritis lesions (**Figure 7B**). Myocardial dysfunction, measured by decreased EF and increased LVID, was detected as early as 2 weeks post-LCWE injection (**Figures 7C, D**), in line with our previous observations (37–39). The decline in myocardial function and LV remodeling persisted and further deteriorated at weeks 12 and 16 post-LCWE injection (**Figures 7C, D**). These results indicate that LCWE-induced KD-like vasculitis, myocarditis, and myocardial fibrosis are also associated with long-term myocardial dysfunction and LV remodeling.

## Discussion

The current study demonstrates that LCWE-induced KD-like vasculitis can lead to long-term cardiovascular sequela, with persistent and severe CAA, dilated abdominal aorta with ongoing inflammatory infiltration, myocarditis, myocardial fibrosis, left ventricular remodeling, and impaired myocardial function. Cardiovascular complications are a significant concern in the long-term management of KD (1). Intense inflammatory cellular infiltrations in the CA destroy the vessel wall leading to aneurysm formation (7, 20, 21, 26, 27). Previously, it was assumed that cardiovascular changes only occurred during the acute phase of the disease. However, histological observations on tissues collected from autopsied KD patients have shown that CAA can develop for several months or even years after diagnosis (17, 19, 30, 31, 41, 42). While CAA may regress after the acute phase, some lesions, especially the larger ones, can persist beyond that period and silently progress to stenosis or form a thrombus (8, 43). Ongoing remodeling in CA abnormalities and injuries can lead to the development of LMP and progressive narrowing and blockage of the CA, which then lead to myocardial ischemia and severe cardiovascular events, such as myocardial infarction (MI) or even sudden death in rare cases, as observed in our experimental murine model. Therefore, it is essential to fully characterize the dynamic pathophysiology of KD to discover novel interventions to prevent or reverse long-term damage.

Since the availability of heart tissues from KD patients is severely limited, the development and use of relevant animal models have been instrumental in better understanding the immune-pathological processes of KD vasculitis. Although no single animal model can genuinely replicate the exact findings of human diseases, including KD vasculitis, experimental animal models mimicking KD pathology [LCWE and *Candida albicans* water-soluble fraction (CAWS)] have proved to be valuable tools for investigating the immunopathological aspects of the disease, provided that limitations of these model systems are acknowledged (5). The LCWE-induced vasculitis animal model exhibits acute neutrophil infiltration, necrotizing vasculitis, aortitis, epicardial coronary arteritis, and myocarditis, closely mimicking the cardiac pathologies observed in human KD patients (5). Additionally, this murine model displays LMP in the healing phase, leading to concentric inflammation and coronary stenosis similar to the reported human KD pathology (5, 19, 21–23). While CA are most studied in patients with KD because of their critical and immediate clinical importance, KD is a widespread vasculitis and may also affect other systemic vessels, including the axillary, subclavian, brachial, renal, and iliac arteries, as well as the abdominal aorta in KD patients (44–47). LCWE injection also induces inflammation in other vessels, such as the abdominal aorta, brachial, iliac, and renal arteries (36, 40, 48–51). In addition, the LCWE-induced murine model of KD also exhibits fever (35), myocardial dysfunction with left ventricular impairment, and EKG changes similar to those reported in human KD patients (38, 39). Notably, this experimental murine model has proven beneficial in translating therapies from preclinical studies to clinical applications (5). Treatments such as IVIG, anti-TNF agents, and anti-IL-1 therapies have demonstrated efficacy in both the animal models and human KD patients (52), with anakinra, an IL-1 receptor antagonist (IL-1Ra), currently undergoing phase III clinical trials (NCT04656184).

Here, we used the LCWE-induced KD-like vasculitis mouse model, which causes systemic vasculitis, aortitis, coronary arteritis, myocarditis, myocardial dysfunction, and myocardial fibrosis in mice (5, 35, 37, 38). LCWE triggers the destruction of elastic layers, promotes intimal hypertrophy, and induces smooth muscle cell (SMC) proliferation towards the lumen, leading to myofibroblast proliferation and CA stenosis, as well as fibrotic remodeling and scarring also reported in children with KD (5, 32, 35, 36, 53). However, since most studies using this experimental model of KD have focused on the acute phase of the disease, it remains unknown whether this model is also useful for evaluating the long-term effects of KD vasculitis. In one of the few studies that assessed later time points, Suganuma et al. observed that maximum intimal thickness and luminal narrowing in the CA occurred 16 weeks after LCWE injection (54). In addition, we previously reported that abdominal aorta aneurysms increase in size (measured as area) within the first 5 weeks of LCWE-injection and plateau and were still present 8 weeks after LCWE injection in this experimental model of KD (36). The current study revealed that LCWE-induced vasculitis can lead to long-lasting cardiovascular lesions that persist for up to 16 weeks, do not regress over time, and leads to 35% mortality at 4 months. While this study does not address the cause of late mortality, we suspect that ongoing remodeling that leads to CA stenosis and myocardial ischemia likely plays a role in the mortality that we have observed. Calcification of the CA may also potentially occur after 16 weeks in LCWE-injected mice. However, this will need to be investigated in future studies extending beyond the 16 weeks of observation.

We observed systemic changes in circulating immune cells and positive correlations between frequencies and numbers of blood neutrophils and inflammatory monocytes during the acute phase of the disease with more severe vasculitis. We also observed an extensive fibrotic area around the aortitis and CA lesions early in the disease process, interstitial fibrosis in the myocardium later in the disease process, and a notable decline in cardiac function early in the disease as previously observed (37–39), which persists up to 16 weeks. Furthermore, there were substantial infiltrations of inflammatory cells in the aortic root and CA, with frequent severe or complete CA stenosis cases up to 16 weeks post-LCWE injection. Interestingly, the evaluation of the heart vessel inflammatory score and development of abdominal aorta aneurysms seemed to plateau around weeks 12 to 16 post-LCWE; however, the cardiovascular lesions did not regress and remained stable. Myocardial fibrosis can also occur in children following KD either due to myocardial ischemia or due to long-term complications of the acute myocarditis that occurs during acute KD (55, 56). Myocarditis occurs frequently during KD (57), and serial myocardial biopsy studies have shown that histological myocarditis develops in most children with KD, even in the absence of CAA or concurrent ischemic damage (42, 58, 59). Myocardial biopsy results indicate ongoing inflammatory cell infiltration and interstitial myocardial fibrosis in patients diagnosed with giant CAA during the acute phase of KD, some with mildly diminished left ventricular EF (60). After conducting follow-up assessments, it was found that 25% of patients had asymptomatic CA obstruction and worsening of fibrosis. Myocardial fibrosis has been associated with myocardial dysfunction and reduced EF (≤55%) amongst KD patients (55). Indeed, KD patients presenting with isolated low EF with normal CA dimensions during the acute phase of their illness may be at risk for developing myocardial fibrosis as a late sequela (55).

Here, we show that LCWE injected mice develop myocarditis starting at week 2 post-injection, which continues up to and including week 12, and that these mice had significantly reduced EF during the acute phase, starting at week 2 post-LCWE and further decreasing over time including at weeks 12 and 16 of follow-up. Myocarditis was observed mostly adjacent to the aortitis and CALs, and not at distal portions of the myocardium. Trichrome staining revealed that the heart undergoes continuous remodeling around the CA circumference, detectable as early as 2 weeks after LCWE injection, peaking on weeks 8 and 12. The entire CA area becomes fibrotic, with no trend to revert to its normal state due to the extension of fibrotic tissue. We also observed diffuse interstitial fibrosis in the myocardium of mice 12 weeks after LCWE injection but not at earlier time points.

The exact mechanism by which LCWE triggers vasculitis is unknown, but it is known to involve innate and adaptive immune responses (5). TLR2 is required to activate LCWE-induced inflammatory response in a MyD88-dependent manner, activating the NF-κB signaling pathway and the production of several inflammatory cytokines, including IL-1β (34). Neutrophils are activated and seem to play an essential role in KD immunopathogenesis (61, 62). When neutrophils are activated, they secret enzymes and proteases, such as neutrophil elastase, to neutralize and eliminate pathogenic threats. However, overactivation and uncontrolled recruitment can lead to tissue damage, destroying the intima and media layers of the CA wall and contributing to lesion formation. In KD, most circulating neutrophils are activated and are a major source of IL-1β (61, 63). Neutrophils infiltrate the media layer of CA during the acute phase of KD (26), and KD patients with giant CAA have increased levels of calprotectin (S100A8–9), which is primarily secreted by neutrophils, years after disease onset (64). However, during the healing stages of KD, approximately two months after disease onset, neutrophils have not been observed in the vessel’s pathology reports (21–23). Here, we observed acute infiltrations of neutrophils in heart tissues of LCWE-injected mice that peaked at 8 weeks post-LCWE and progressively declined over time. The presence of remaining neutrophilic infiltrates around the CAs up to 16 weeks post-LCWE injection in this animal model is a discordant finding from published human pathological studies reporting absence of neutrophils in the later stages of KD (19, 21–23). Alterations in the peripheral levels of neutrophils have been reported during KD, and neutrophilia occurs during the acute and subacute phases of the disease (62, 63, 65). LCWE-injected mice also exhibited increased circulating counts and percentages of leukocytes and neutrophils in peripheral blood. We have also established a clear correlation between the severity of abdominal aorta dilations and heart vessel inflammation with frequencies and counts of circulating neutrophils during the acute phase of LCWE-induced KD-like vasculitis. Human monocytes can be classified as classical (CD14^+^ CD16^-^), intermediate (CD14^+^ CD16^+^), and nonclassical (CD14^+^ CD16^++^) monocytes (66). Intermediate CD14^+^ CD16^+^ monocyte counts and frequencies are elevated during the acute phase of KD when compared with convalescent IVIG-treated KD patients and healthy control subjects (67). A higher proportion of classical monocytes is also reported during acute KD (68). Moreover, increased peripheral CD14^+^ monocyte/macrophage levels were found in patients with CAA compared with KD patients without lesions, and the absolute counts of CD14^+^ macrophages/monocytes correlated with the severity of vascular damage during acute KD (69, 70). In addition, treatment with IVIG appears to rapidly decrease the levels of monocytes in IVIG-responsive KD patients (67). Furthermore, not only the frequencies of circulating CD16^+^ monocytes are increased in KD patients compared to IVIG-treated convalescent KD patients, but these cells also express higher levels of pro-inflammatory mediators, including *TNF*, *IL1B*, and S100 genes (71). Our flow cytometric analysis revealed increased numbers of circulating inflammatory, patrolling, and intermediate monocytes during the acute phase of LCWE-induced KD-like vasculitis, which correlated with the severity of the abdominal aorta dilation and aneurysms. We also observed a reduction in the number of circulating monocytes in the long-term phase of the disease, reaching levels almost similar to the ones observed in PBS control mice. Our observations in the LCWE mouse model of KD-like vasculitis, associated with the ones reported in KD patients, suggest a key role of monocytes during the acute phase of the disease.

A better understanding of the immunopathology of the late complications of KD related to myocarditis and myocardial fibrosis and remaining long-term inflammation in CA is needed. Our study demonstrates that LCWE-induced KD-like vasculitis can lead to long-term histopathological changes, continuous cardiovascular inflammation, diminished ventricular function, left ventricular remodeling, and myocardial fibrosis severely impacting cardiac function for up to 16 weeks, similar to the long-term effects observed in patients with KD. Therefore, the murine model of LCWE-induced KD-like vasculitis may be suitable for investigating the molecular mechanisms and immunopathology of the long-term consequences of KD vasculitis, which may lead to the development of novel strategies to prevent and treat the late cardiovascular complications of the disease.

## Materials and methods

### Mice

Five-week-old, Wild-Type (WT) C57BL/6 male mice were purchased from the Jackson Laboratory (Bar Harbor, ME, USA). WT mice were randomly assigned to each experimental group. Mice were housed under specific pathogen-free conditions and used according to the Cedars-Sinai Medical Center (CSMC) institutional committee guidelines. All animal studies were approved by the Institutional Animal Care and Use Committee at Cedars-Sinai Medical Center and performed following the Guide for the Care and Use of Laboratory Animals published by the U.S. National Institutes of Health.

### LCWE-induced KD-like vasculitis mouse model

*Lactobacillus casei* (ATCC 11578) cell wall extract (LCWE) was prepared as previously described (35). Briefly, *Lactobacillus casei* was grown in Man-Rogosa-Sharpe broth (Sigma-Aldrich, #1.10661) for 48 hours in 37°C incubation, harvested and washed with PBS (1:1 volume). Harvested bacteria were spun down and resuspended in 20 ml of PBS for every 5 grams of bacteria pellet, followed by 2 hours of sonication using a 3/4-inch horn and a garnet tip at maximum power. Samples were maintained in a dry ice/ethanol bath during the sonication procedure to prevent overheating. After sonication, bacteria were centrifuged for 20 minutes at 12000 rpm and 4°C. The supernatant was obtained and centrifuged for 1 hour at 38000 rpm and 4°C. The final supernatant was collected and stored at -80°C. Five-week-old WT male mice were injected with a single dose of 500 µl of either LCWE or PBS intraperitoneally (i.p.) to induce systemic vasculitis, as previously published (33–40, 48–51, 53, 72). At weeks 2, 4, 8, 12, and 16 post-LCWE injection, blood was collected, and mice were euthanized. Mice were then perfused with PBS, and heart tissues were collected and embedded in a tissue-tek optimum cutting temperature (O.C.T.) compound (Sakura Finetek, catalog #4583). Abdominal aortas were dissected from the level of the left renal artery down to the iliac bifurcation, photographed, and embedded in O.C.T., as previously published (36, 40, 48–51, 72). The maximal abdominal aorta diameter was determined by measuring five different areas separated by 2 mm of the abdominal aorta infra-renal portion (below the left renal artery) with ImageJ (NIH) (36, 40, 48–51, 72). The infrarenal abdominal aorta area was also measured in ImageJ (36, 48–51, 72). Heart tissue sections were stained with Masson’s trichrome (Millipore Sigma, catalog #HT15), hematoxylin, and eosin (H&E; Millipore Sigma, catalog #MHS32). Sections were visualized and captured on a Keyence’s BZ-9000 microscope, with the BZ-II viewer and BZ-II analyzer software (Keyence). Heart tissue histopathological examination and assessment of the severity of cardiovascular lesions (CAs, aortic root vasculitis, and myocarditis) were performed on H&E-stained tissue sections, and given a heart vessel severity score by an expert pathologist blinded to the experimental groups, as previously described (35, 72). Briefly, acute inflammation, chronic inflammation, and connective tissue proliferation were each assessed using the following scoring system: 0 = no inflammation, 1 = rare inflammatory cells, 2 = scattered inflammatory cells, 3 = diffuse infiltrate of inflammatory cells, and 4 = dense clusters of inflammatory cells. Fibrosis was determined using the following scoring system: 0 = no medial fibrosis, 1 = medial fibrosis involving less than 10% of the CA circumference, 2 = medial fibrosis involving 11% to 50% of the CA circumference, 3 = medial fibrosis involving 51% to 75% of the CA circumference, and 4 = medial fibrosis involving more than 75% of the CA circumference. As previously published, all four scores were combined to generate a severity score over 16 called the “Heart inflammation score” (35, 40, 48–50, 72). Myocardial inflammation score (myocarditis score) was described previously (35). Briefly, 0 = no myocardial inflammation and myocardial fibrosis, 1 = rare inflammatory cells in myocardium or very minimal focal subepicardial interstitial fibrosis just infiltrating beneath epicardial fat, 2 = scattered inflammatory cells in myocardium, and/or mild subepicardial interstitial fibrosis infiltrating deeper into the subepicardial myocardium, 3 = diffuse inflammatory cells in myocardium and/or multifocal subepicardial interstitial fibrosis, and 4 = dense inflammatory cells in myocardium and/or replacement fibrosis.

### Peripheral blood cell isolation

Mice were anesthetized with isoflurane, and blood was collected by retro-orbital bleeding using a heparinized micro-hematocrit capillary tube (Fisher Scientific, #22–362-566). 40 μl of blood was transferred to a 1.5 ml tube, and red blood cell lysis was performed by adding 200 μl of RBC lysis buffer (eBioscience, catalog #00–4333-57). After 2 minutes, 500 μl of wash buffer (1X PBS and 1% FBS) was added, and samples were centrifuged at 2000 rpm for 5 minutes at 4°C. Pellets were suspended in 50 μl of wash buffer and stained for flow cytometric analysis.

### Flow cytometric analysis

Samples were first incubated with an anti-murine CD16/CD32 antibody (Clone 2.4G2; Tonbo Biosciences, catalog #70–0161-M001) for 10 minutes. The following murine antibodies were used for flow cytometric analysis: CD45.2 (1 µg/ml, AF700, clone 104, Biolegend, catalog #109822), CD11b (1 µg/ml, VioletFluor 450, clone M1/70, Tonbo Bioscience, catalog #75–0112), Ly6G (1 µg/ml, APC, clone 1A8, Tonbo Bioscience, catalog # 20–1276) or Ly6C (1 µg/ml, PE, clone HK1.4, Biolegend, catalog #128008). Dead cells were excluded using a fixable viability dye (eFluor 506, Invitrogen, catalog #65–0866-14). Samples were incubated on ice for 30 minutes with the antibodies, then washed two times with buffer (1X PBS and 1% FBS) and fixed with 0.5% paraformaldehyde (PFA). Before sample acquisition, counting beads were added to each sample (CountBright, Life Technologies, catalog #C36950). Data was acquired on a SONY 3800 Spectral Cell Analyzer (Sony Biotechnology) and analyzed using FlowJo Software (BD Bioscience).

### Immunofluorescence

Tissues were embedded in O.C.T. compound and frozen in 2-methylbutane precooled in liquid nitrogen, then stored at –80°C until sectioning. Serial sections of the heart were cut at the aortic root level. Heart and abdominal aorta tissue cryosections (7 μm) collected from mice injected with either PBS or LCWE were fixed in cold acetone for 5 minutes, washed in PBS, and blocked for 1 hour with anti-goat serum. Samples were stained overnight with the following antibodies: Ly6G (Clone 1A8, BioLegend, catalog #127610), CD3 (Cell Signaling, catalog #78588S). Isotype controls were used as negative controls: Rat IgG2 (Biolegend, catalog #ab400526) and Rabbit IgG (Abcam, catalog #1ab71870). After washing three times with PBS, sections were mounted with DAPI (Abcam, catalog #ab104139). Images were obtained with a Keyence BZ-9000 fluorescence microscope.

### Transthoracic echocardiogram

Transthoracic echocardiography was performed using a VisualSonics Vevo 3100 system equipped with a MX550D transducer (Visual Sonics) (73–75). Left ventricular ejection fraction (EF) and dimensions were obtained from short-axis M-mode scans at the midventricular level. Mice were anesthetized with 4% isoflurane (induction) and maintained with 2–1% isoflurane on a body-temperature-controlled pad. Echocardiogram acquisition was initiated when the heart rate ranged between 420 to 500 beats per min. Measurements were obtained on 4 or more consecutive cardiac cycles by tracing the left ventricular free walls and averaged for each mouse. All the image analyses were conducted by an independent trained observer blinded to the experimental groups.

### Statistical analysis

Pooled data are presented as mean ± SEM. The normality of data was assessed using the Shapiro-Wilk test. A one-way analysis of variance (ANOVA) was used for multiple comparisons. Tukey post-test analysis was used for normally distributed data, and the Kruskal Wallis test with Dunn’s multiple comparisons was used for non-normally distributed data. A comparison of Kaplan-Meier survival analysis was done using the log-rank (Mantel-Cox) test. Spearman’s correlation test was used to analyze two variables. A value of p<0.05 was considered statistically significant. Data were analyzed using GraphPad Prism Software (version 10).

## Data availability statement

The raw data supporting the conclusions of this article will be made available by the authors, without undue reservation.

## Ethics statement

The animal study was approved by the Institutional Animal Care and Use Committee at Cedars-Sinai Medical Center. The study was conducted in accordance with the local legislation and institutional requirements.

## Author contributions

ALP: Conceptualization, Formal analysis, Investigation, Methodology, Visualization, Writing – original draft, Writing – review & editing. EA: Investigation, Methodology, Writing – original draft. DM: Investigation, Writing – original draft, Methodology. ML: Investigation, Writing – original draft, Methodology. TTC: Writing – original draft, Formal analysis, Investigation, Methodology. TM: Formal analysis, Investigation, Writing – original draft. YL: Investigation, Writing – original draft. TC: Investigation, Writing – original draft, Formal analysis, Supervision, Writing – review & editing. RP: Formal analysis, Investigation, Supervision, Writing – original draft, Conceptualization, Methodology. WV: Formal analysis, Investigation, Supervision, Writing – original draft. MNR: Formal analysis, Investigation, Supervision, Writing – original draft, Conceptualization, Funding acquisition, Resources, Visualization, Writing – review & editing. MA: Writing – original draft, Writing – review & editing, Conceptualization, Formal analysis, Funding acquisition, Investigation, Resources, Supervision, Visualization.

## References

[1] McCrindle BW Rowley AH Newburger JW Burns JC Bolger AF Gewitz M. Diagnosis, treatment, and long-term management of kawasaki disease. Circulation. (2017) 135:e927–e99. doi: 10.1161/CIR.0000000000000484 28356445

[2] Soni PR Noval Rivas M Arditi M. A comprehensive update on kawasaki disease vasculitis and myocarditis. Curr Rheumatol Rep. (2020) 22:6. doi: 10.1007/s11926-020-0882-1 32020498

[3] Rowley AH. Kawasaki disease: novel insights into etiology and genetic susceptibility. Annu Rev Med. (2011) 62:69–77. doi: 10.1146/annurev-med-042409-151944 20690826 PMC3021097

[4] Rowley AH. Is kawasaki disease an infectious disorder? Int J Rheum Dis. (2018) 21:20–5. doi: 10.1111/1756-185x.13213 PMC5777874 29105346

[5] Noval Rivas M Arditi M. Kawasaki disease: pathophysiology and insights from mouse models. Nat Rev Rheumatol. (2020) 16:391–405. doi: 10.1038/s41584-020-0426-0 32457494 PMC7250272

[6] Kim GB. Reality of kawasaki disease epidemiology. Korean J Pediatr. (2019) 62:292–6. doi: 10.3345/kjp.2019.00157 PMC6702118 31319643

[7] Akagi T Rose V Benson LN Newman A Freedom RM. Outcome of coronary artery aneurysms after kawasaki disease. J Pediatr. (1992) 121:689–94. doi: 10.1016/s0022-3476(05)81894-3 1432415

[8] Friedman KG Gauvreau K Hamaoka-Okamoto A Tang A Berry E Tremoulet AH. Coronary artery aneurysms in kawasaki disease: risk factors for progressive disease and adverse cardiac events in the us population. J Am Heart Assoc. (2016) 5(9):e003289. doi: 10.1161/jaha.116.003289 27633390 PMC5079009

[9] Kato H Ichinose E Kawasaki T. Myocardial infarction in kawasaki disease: clinical analyses in 195 cases. J Pediatr. (1986) 108:923–7. doi: 10.1016/s0022-3476(86)80928-3 3712157

[10] McCrindle BW Li JS Minich LL Colan SD Atz AM Takahashi M. Coronary artery involvement in children with kawasaki disease: risk factors from analysis of serial normalized measurements. Circulation. (2007) 116:174–9. doi: 10.1161/circulationaha.107.690875 17576863

[11] Phuong LK Curtis N Gowdie P Akikusa J Burgner D. Treatment options for resistant kawasaki disease. Paediatr Drugs. (2018) 20:59–80. doi: 10.1007/s40272-017-0269-6 29101553

[12] Lo MS Newburger JW. Role of intravenous immunoglobulin in the treatment of kawasaki disease. Int J Rheum Dis. (2018) 21:64–9. doi: 10.1111/1756-185x.13220 29205910

[13] Tremoulet AH Best BM Song S Wang S Corinaldesi E Eichenfield JR. Resistance to intravenous immunoglobulin in children with kawasaki disease. J Pediatr. (2008) 153:117–21. doi: 10.1016/j.jpeds.2007.12.021 PMC2526555 18571548

[14] Skochko SM Jain S Sun X Sivilay N Kanegaye JT Pancheri J. Kawasaki disease outcomes and response to therapy in a multiethnic community: A 10-year experience. J Pediatr. (2018) 203:408–15.e3. doi: 10.1016/j.jpeds.2018.07.090 30268398

[15] Gordon JB Daniels LB Kahn AM Jimenez-Fernandez S Vejar M Numano F. The spectrum of cardiovascular lesions requiring intervention in adults after kawasaki disease. JACC Cardiovasc Interv. (2016) 9:687–96. doi: 10.1016/j.jcin.2015.12.011 27056307

[16] Gordon JB Kahn AM Burns JC. When children with kawasaki disease grow up: myocardial and vascular complications in adulthood. J Am Coll Cardiol. (2009) 54:1911–20. doi: 10.1016/j.jacc.2009.04.102 PMC2870533 19909870

[17] Kato H Sugimura T Akagi T Sato N Hashino K Maeno Y. Long-term consequences of kawasaki disease. A 10- to 21-year follow-up study of 594 patients. Circulation. (1996) 94:1379–85. doi: 10.1161/01.CIR.94.6.1379 8822996

[18] Rizk SR El Said G Daniels LB Burns JC El Said H Sorour KA. Acute myocardial ischemia in adults secondary to missed kawasaki disease in childhood. Am J Cardiol. (2015) 115:423–7. doi: 10.1016/j.amjcard.2014.11.024 PMC4697961 25555655

[19] Orenstein JM Shulman ST Fox LM Baker SC Takahashi M Bhatti TR. Three linked vasculopathic processes characterize kawasaki disease: A light and transmission electron microscopic study. PloS One. (2012) 7:e38998. doi: 10.1371/journal.pone.0038998 22723916 PMC3377625

[20] Shulman ST Rowley AH. Kawasaki disease: insights into pathogenesis and approaches to treatment. Nat Rev Rheumatol. (2015) 11:475–82. doi: 10.1038/nrrheum.2015.54 25907703

[21] Takahashi K Oharaseki T Yokouchi Y. Histopathological aspects of cardiovascular lesions in kawasaki disease. Int J Rheum Dis. (2017) 21:31–5. doi: 10.1111/1756-185X.13207 29105353

[22] Fujiwara H Fujiwara T Kao TC Ohshio G Hamashima Y. Pathology of kawasaki disease in the healed stage. Relationships between typical and atypical cases of kawasaki disease. Acta Pathol Jpn. (1986) 36:857–67. doi: 10.1111/j.1440-1827.1986.tb03119.x 3766134

[23] Hamashima Y. Kawasaki disease. Heart Vessels Suppl. (1985) 1:271–6. doi: 10.1007/bf02072407 3843587

[24] Rowley AH Shulman ST Mask CA Finn LS Terai M Baker SC. Iga plasma cell infiltration of proximal respiratory tract, pancreas, kidney, and coronary artery in acute kawasaki disease. J Infect Dis. (2000) 182:1183–91. doi: 10.1086/315832 10979916

[25] Brown TJ Crawford SE Cornwall ML Garcia F Shulman ST Rowley AH. Cd8 T lymphocytes and macrophages infiltrate coronary artery aneurysms in acute kawasaki disease. J Infect Dis. (2001) 184:940–3. doi: 10.1086/323155 11528596

[26] Takahashi K Oharaseki T Naoe S Wakayama M Yokouchi Y. Neutrophilic involvement in the damage to coronary arteries in acute stage of kawasaki disease. Pediatr Int. (2005) 47:305–10. doi: 10.1111/j.1442-200x.2005.02049.x 15910456

[27] Takahashi K Oharaseki T Yokouchi Y. Pathogenesis of kawasaki disease. Clin Exp Immunol. (2011) 164 Suppl 1:20–2. doi: 10.1111/j.1365-2249.2011.04361.x PMC3095860 21447126

[28] Robinson C Chanchlani R Gayowsky A Brar S Darling E Demers C. Cardiovascular outcomes in children with kawasaki disease: A population-based cohort study. Pediatr Res. (2023) 93:1267–75. doi: 10.1038/s41390-022-02391-3 36380069

[29] De Rosa G Andreozzi L Piastra M Castelli B Rigante D. Acute myocarditis as a revealing clue of complete kawasaki disease. Reumatismo. (2018) 70:115–6. doi: 10.4081/reumatismo.2018.1101 29976047

[30] Anderson TM Meyer RA Kaplan S. Long-term echocardiographic evaluation of cardiac size and function in patients with kawasaki disease. Am Heart J. (1985) 110(1 Pt 1):107–15. doi: 10.1016/0002-8703(85)90523-x 3925739

[31] Burns JC Shike H Gordon JB Malhotra A Schoenwetter M Kawasaki T. Sequelae of kawasaki disease in adolescents and young adults. J Am Coll Cardiol. (1996) 28:253–7. doi: 10.1016/0735-1097(96)00099-X 8752822

[32] Lehman TJ Walker SM Mahnovski V McCurdy D. Coronary arteritis in mice following the systemic injection of group B lactobacillus casei cell walls in aqueous suspension. Arthritis Rheum. (1985) 28:652–9. doi: 10.1002/art.1780280609 3924060

[33] Abe M Rastelli DD Gomez AC Cingolani E Lee Y Soni PR. Il-1-dependent electrophysiological changes and cardiac neural remodeling in a mouse model of kawasaki disease vasculitis. Clin Exp Immunol. (2019) 199(3):303–13. doi: 10.1111/cei.13401 PMC7008220 31758701

[34] Rosenkranz ME Schulte DJ Agle LMA Wong MH Zhang W Ivashkiv L. Tlr2 and myd88 contribute to lactobacillus casei extract-induced focal coronary arteritis in a mouse model of kawasaki disease. Circulation. (2005) 112:2966–73. doi: 10.1161/CIRCULATIONAHA.105.537530 16275884

[35] Lee Y Schulte DJ Shimada K Chen S Crother TR Chiba N. Interleukin-1β Is crucial for the induction of coronary artery inflammation in a mouse model of kawasaki disease. Circulation. (2012) 125:1542–50. doi: 10.1161/circulationaha.111.072769 PMC3337219 22361326

[36] Wakita D Kurashima Y Crother TR Noval Rivas M Lee Y Chen S. Role of interleukin-1 signaling in a mouse model of kawasaki disease-associated abdominal aortic aneurysm. Arterioscler Thromb Vasc Biol. (2016) 36:886–97. doi: 10.1161/atvbaha.115.307072 PMC4850105 26941015

[37] Mesquita T Lin YN Chen S Lee Y Miguel-Dos-Santos R Atici AE. Inhibition of il-1 ameliorates cardiac dysfunction and arrhythmias in a murine model of kawasaki disease. Arterioscler Thromb Vasc Biol. (2024) 44(4):e117–30. doi: 10.1161/atvbaha.123.320382 PMC10978283 38385289

[38] Matundan HH Sin J Rivas MN Fishbein MC Lehman TJ Chen S. Myocardial fibrosis after adrenergic stimulation as a long-term sequela in a mouse model of kawasaki disease vasculitis. JCI Insight. (2019) 4:e126279. doi: 10.1172/jci.insight.126279 30728329 PMC6413776

[39] Gorelik M Lee Y Abe M Andrews T Davis L Patterson J. Il-1 receptor antagonist, anakinra, prevents myocardial dysfunction in a mouse model of kawasaki disease vasculitis and myocarditis. Clin Exp Immunol. (2019) 198(1):101–10. doi: 10.1111/cei.13314 PMC6718290 31099056

[40] Noval Rivas M Wakita D Franklin MK Carvalho TT Abolhesn A Gomez AC. Intestinal permeability and iga provoke immune vasculitis linked to cardiovascular inflammation. Immunity. (2019) 51:508–21.e6. doi: 10.1016/j.immuni.2019.05.021 31471109 PMC6751009

[41] Yonesaka S Nakada T Sunagawa Y Tomimoto K Naka S Takahashi T. Endomyocardial biopsy in children with kawasaki disease. Acta Paediatr Jpn. (1989) 31:706–11. doi: 10.1111/j.1442-200X.1989.tb01384.x 2516398

[42] Yutani C Go S Kamiya T Hirose O Misawa H Maeda H. Cardiac biopsy of kawasaki disease. Arch Pathol Lab Med. (1981) 105(9):470–3. 6895017

[43] McCrindle BW Manlhiot C Newburger JW Harahsheh AS Giglia TM Dallaire F. Medium-term complications associated with coronary artery aneurysms after kawasaki disease: A study from the international kawasaki disease registry. J Am Heart Assoc. (2020) 9:e016440. doi: 10.1161/jaha.119.016440 32750313 PMC7792232

[44] Amano S Hazama F Kubagawa H Tasaka K Haebara H Hamashima Y. General pathology of kawasaki disease. On the morphological alterations corresponding to the clinical manifestations. Acta pathologica japonica. (1980) 30:681–94. doi: 10.1111/j.1440-1827.1980.tb00966.x 7446109

[45] Hoshino S Tsuda E Yamada O. Characteristics and fate of systemic artery aneurysm after kawasaki disease. J Pediatr. (2015) 167(1):108–12.e1–2. doi: 10.1016/j.jpeds.2015.04.036 25981909

[46] Shobun T Kyung C Madeline M Samuel G Stanford TS. Peripheral gangrene associated with kawasaki disease. Clin Infect Dis. (1992) 14:121–6. doi: 10.1093/clinids/14.1.121 1571415

[47] Miyake T Yokoyama T Shinohara T Seto S Oiki M. Transient dilatation of the abdominal aorta in an infant with kawasaki disease associated with thrombocytopenia. Acta Paediatr Jpn. (1995) 37:521–5. doi: 10.1111/j.1442-200X.1995.tb03368.x 7572158

[48] Marek-Iannucci S Ozdemir AB Moreira D Gomez AC Lane M Porritt RA. Autophagy-mitophagy induction attenuates cardiovascular inflammation in a murine model of kawasaki disease vasculitis. JCI Insight. (2021) 6(18):e151981. doi: 10.1172/jci.insight.151981 34403365 PMC8492304

[49] Marek-Iannucci S Yildirim AD Hamid SM Ozdemir AB Gomez AC Kocatürk B. Targeting ire1 endoribonuclease activity alleviates cardiovascular lesions in a murine model of kawasaki disease vasculitis. JCI Insight. (2022) 7(6):e157203. doi: 10.1172/jci.insight.157203 35167493 PMC8986066

[50] Porritt RA Markman JL Maruyama D Kocaturk B Chen S Lehman TJA. Interleukin-1 beta-mediated sex differences in kawasaki disease vasculitis development and response to treatment. Arterioscler Thromb Vasc Biol. (2020) 40:802–18. doi: 10.1161/ATVBAHA.119.313863 PMC7047651 31996019

[51] Porritt RA Zemmour D Abe M Lee Y Narayanan M Carvalho TT. Nlrp3 inflammasome mediates immune-stromal interactions in vasculitis. Circ Res. (2021) 129:e183–200. doi: 10.1161/circresaha.121.319153 PMC8555446 34517723

[52] Burns JC Kone-Paut I Kuijpers T Shimizu C Tremoulet A Arditi M. Review: found in translation: international initiatives pursuing interleukin-1 blockade for treatment of acute kawasaki disease. Arthritis Rheumatol. (2017) 69:268–76. doi: 10.1002/art.39975 PMC5274552 27792871

[53] Lee Y Wakita D Dagvadorj J Shimada K Chen S Huang G. Il-1 signaling is critically required in stromal cells in kawasaki disease vasculitis mouse model: role of both il-1alpha and il-1beta. Arterioscler Thromb Vasc Biol. (2015) 35:2605–16. doi: 10.1161/atvbaha.115.306475 PMC4662607 26515418

[54] Suganuma E Sato S Honda S Nakazawa A. All trans retinoic acid alleviates coronary stenosis by regulating smooth muscle cell function in a mouse model of kawasaki disease. Sci Rep. (2021) 11:13856. doi: 10.1038/s41598-021-93459-3 34226641 PMC8257698

[55] Hoshino S Shimizu C Jain S He F Tremoulet AH Burns JC. Biomarkers of inflammation and fibrosis in kawasaki disease patients years after initial presentation with low ejection fraction. J Am Heart Assoc. (2020) 9:e014569. doi: 10.1161/jaha.119.014569 31880981 PMC6988139

[56] Dusenbery SM Newburger JW Colan SD Gauvreau K Baker A Powell AJ. Myocardial fibrosis in patients with a history of kawasaki disease. Int J Cardiol Heart Vasc. (2021) 32:100713. doi: 10.1016/j.ijcha.2021.100713 33521237 PMC7820031

[57] Dionne A Dahdah N. Myocarditis and kawasaki disease. Int J Rheum Dis. (2018) 21:45–9. doi: 10.1111/1756-185x.13219 29105303

[58] Takahashi M. Myocarditis in kawasaki syndrome. A Minor Villain? Circ. (1989) 79:1398–400. doi: 10.1161/01.cir.79.6.1398 2720935

[59] Harada M Yokouchi Y Oharaseki T Matsui K Tobayama H Tanaka N. Histopathological characteristics of myocarditis in acute-phase kawasaki disease. Histopathology. (2012) 61:1156–67. doi: 10.1111/j.1365-2559.2012.04332.x 23134515

[60] Yonesaka S Takahashi T Eto S Sato T Otani K Ueda T. Biopsy-proven myocardial sequels in kawasaki disease with giant coronary aneurysms. Cardiol Young. (2010) 20:602–9. doi: 10.1017/s1047951109991132 20584347

[61] Beltran JVB Lin FP Chang CL Ko TM. Single-cell meta-analysis of neutrophil activation in kawasaki disease and multisystem inflammatory syndrome in children reveals potential shared immunological drivers. Circulation. (2023) 148:1778–96. doi: 10.1161/circulationaha.123.064734 37905415

[62] Takeshita S KY Kanai T Yoshida Y Tsujita Y. The role of neutrophil activation in the pathogenesis of kawasaki disease. Pediatr Infect Dis. (2018) 3(1):1. doi: 10.4172/2573-0282.100057

[63] Zhu YP Shamie I Lee JC Nowell CJ Peng W Angulo S. Immune response to intravenous immunoglobulin in patients with kawasaki disease and mis-C. J Clin Invest. (2021) 131(20):e147076. doi: 10.1172/jci147076 34464357 PMC8516453

[64] Lech M Guess J Duffner J Oyamada J Shimizu C Hoshino S. Circulating markers of inflammation persist in children and adults with giant aneurysms after kawasaki disease. Circ Genom Precis Med. (2019) 12:e002433. doi: 10.1161/circgen.118.002433 30844302

[65] Biezeveld MH van Mierlo G Lutter R Kuipers IM Dekker T Hack CE. Sustained activation of neutrophils in the course of kawasaki disease: an association with matrix metalloproteinases. Clin Exp Immunol. (2005) 141:183–8. doi: 10.1111/j.1365-2249.2005.02829.x PMC1809423 15958085

[66] Cormican S Griffin MD. Human monocyte subset distinctions and function: insights from gene expression analysis. Front Immunol. (2020) 11:1070. doi: 10.3389/fimmu.2020.01070 32582174 PMC7287163

[67] Katayama K Matsubara T Fujiwara M Koga M Furukawa S. Cd14+Cd16+ Monocyte subpopulation in kawasaki disease. Clin Exp Immunol. (2000) 121:566–70. doi: 10.1046/j.1365-2249.2000.01321.x PMC1905728 10971526

[68] Kim YS Yang HJ Kee SJ Choi I Ha K Ki KK. The "Intermediate" Cd14 + Cd16 + Monocyte subpopulation plays a role in ivig responsiveness of children with kawasaki disease. Pediatr Rheumatol Online J. (2021) 19:76. doi: 10.1186/s12969-021-00573-7 34059085 PMC8165978

[69] Furukawa S Matsubara T Yabuta K. Mononuclear cell subsets and coronary artery lesions in kawasaki disease. Arch Dis Child. (1992) 67:706–8. doi: 10.1136/adc.67.6.706 PMC1793803 1378258

[70] Furukawa S Matsubara T Jujoh K Sasai K Nakachi S Sugawara T. Reduction of peripheral blood macrophages/monocytes in kawasaki disease by intravenous gammaglobulin. Eur J Pediatr. (1990) 150:43–7. doi: 10.1007/bf01959479 1706665

[71] Wang Z Xie L Ding G Song S Chen L Li G. Single-cell rna sequencing of peripheral blood mononuclear cells from acute kawasaki disease patients. Nat Commun. (2021) 12:5444. doi: 10.1038/s41467-021-25771-5 34521850 PMC8440575

[72] Kocatürk B Lee Y Nosaka N Abe M Martinon D Lane ME. Platelets exacerbate cardiovascular inflammation in a murine model of kawasaki disease vasculitis. JCI Insight. (2023) 8(14):e169855. doi: 10.1172/jci.insight.169855 37279077 PMC10443810

[73] Mesquita TRR Zhang R de Couto G Valle J Sanchez L Rogers RG. Mechanisms of atrial fibrillation in aged rats with heart failure with preserved ejection fraction. Heart Rhythm. (2020) 17:1025–33. doi: 10.1016/j.hrthm.2020.02.007 PMC7802887 32068183

[74] Mesquita T Zhang R Cho JH Zhang R Lin YN Sanchez L. Mechanisms of sinoatrial node dysfunction in heart failure with preserved ejection fraction. Circulation. (2022) 145:45–60. doi: 10.1161/circulationaha.121.054976 34905696 PMC9083886

[75] Lin YN Mesquita T Sanchez L Chen YH Liu W Li C. Extracellular vesicles from immortalized cardiosphere-derived cells attenuate arrhythmogenic cardiomyopathy in desmoglein-2 mutant mice. Eur Heart J. (2021) 42:3558–71. doi: 10.1093/eurheartj/ehab419 PMC8442111 34345905

