# Supplementary material for: Long-term cardiovascular inflammation and fibrosis in a murine model of vasculitis induced by Lactobacillus casei cell wall extract
Source: Front Immunol. 2024 Jun 25;15:1411979. doi: 10.3389/fimmu.2024.1411979 (PMC11234797; doi:10.3389/fimmu.2024.1411979)
Supplement: Supplementary file 1 [file DataSheet_1.pdf]

Supplementary Figure S1

A

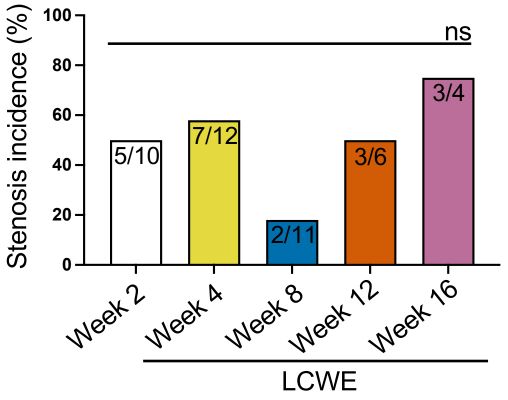

B

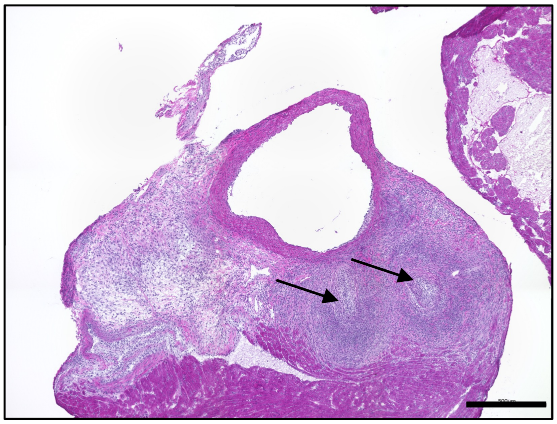

LCWE WEEK 4

Supplementary Figure S1. Stenosis frequency in the coronary arteries up to 16 weeks after LCWE injection. (A) Frequency of coronary artery complete stenosis at different time points post-LCWE injection. **(B)** Representative picture of an H&E-stained heart tissue section showing complete stenosis at 4 weeks post-LCWE injection. Arrows indicate CA occlusion. Scale bar: 500 μm. One-way ANOVA with Tukey post-test (n= 4-12 per group). CA; coronary artery.

Supplementary Figure S2

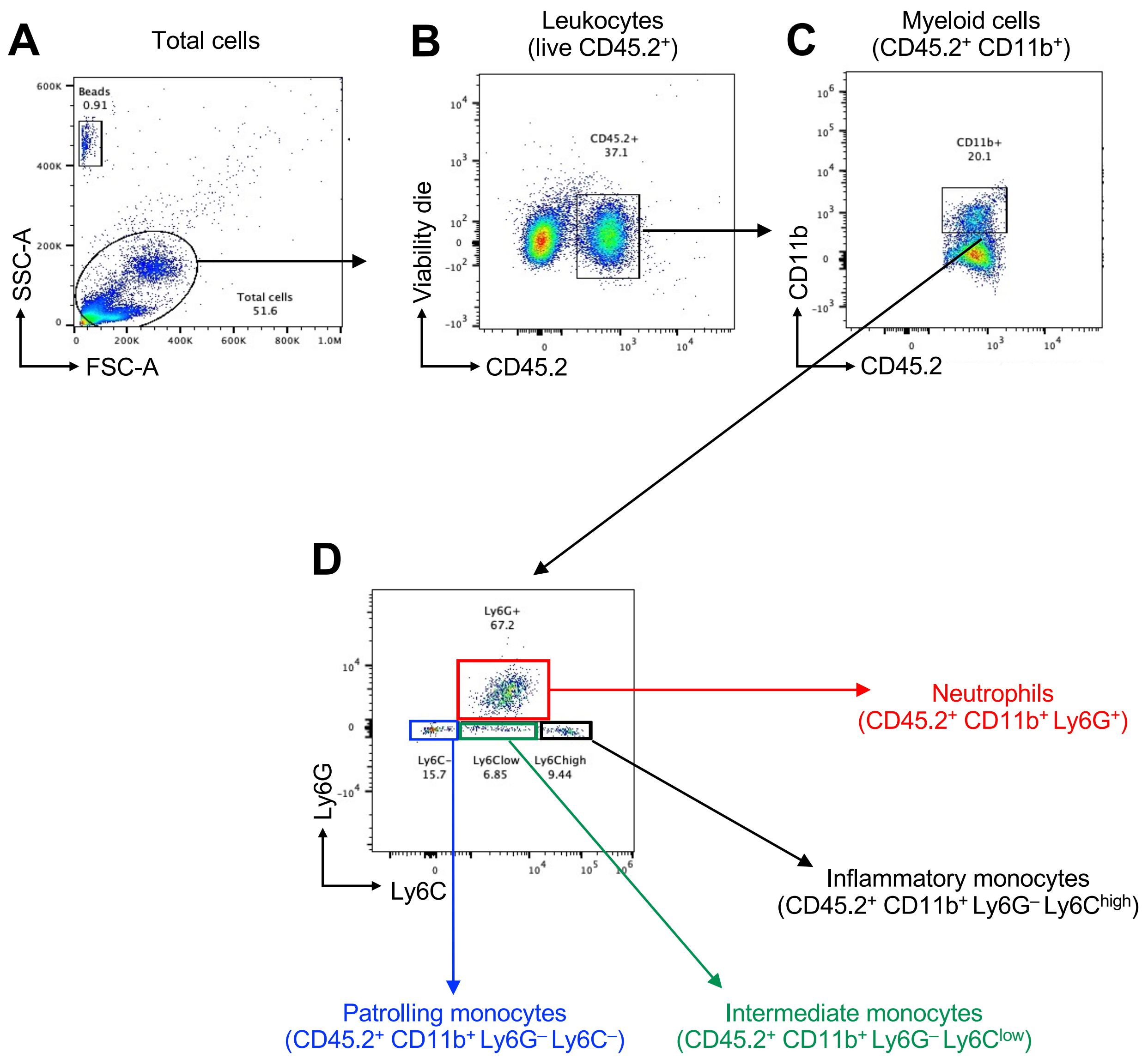

Supplementary Figure S2. Flow cytometric gating strategy used for the analysis of peripheral blood. (A-D) Representative flow plots showing the gating strategy used to characterize and determine the frequencies of leukocytes (B), myeloid cells (C), neutrophils (D), inflammatory monocytes (D), patrolling monocytes (D), and intermediate monocytes (D) in the blood of PBS and LCWE-injected mice.

Supplementary Figure S3

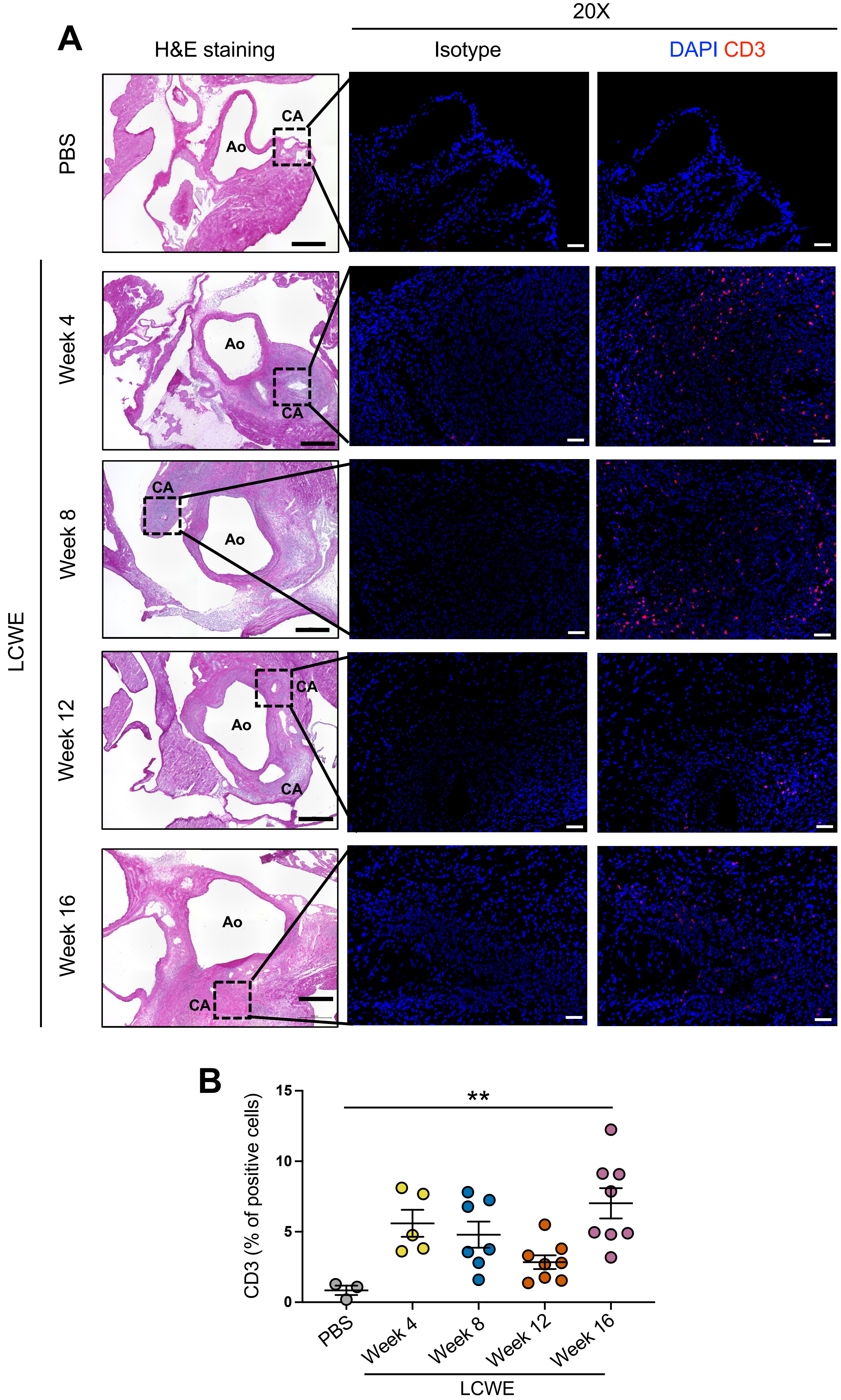

Supplementary Figure S3. Long-term T cell infiltrations in heart tissues of LCWE-injected mice. (A) Representative H&E images and CD3 immunofluorescent (IF) staining (red) in heart tissues from PBS and LCWE-injected mice at different time points post-LCWE injection (n= 3-8 per group). DAPI (blue) was used to identify cell nuclei. **(B)** Quantification of positive CD3 cells in heart tissues from PBS and LCWE-injected mice at different time points post-LCWE injection (n=3-8 per group). The result is expressed in the percentage of CD3 cells out of DAPI within the heart section. Scale bar in H&E images: 500 μm. Scale bar for 20X images: 50 μm. Data are presented as mean ± SEM. ***p*<0.01, by one-way ANOVA with Tukey post-tests. CA, coronary artery; Ao; aorta

Supplementary Figure S4

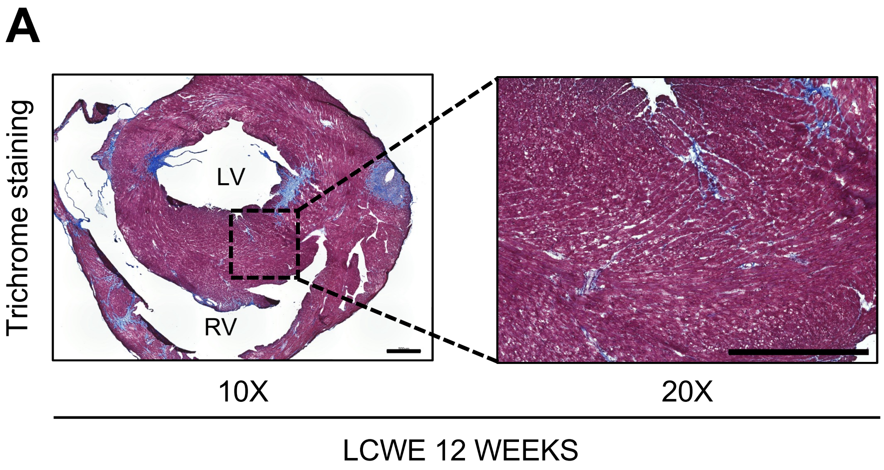

Supplementary Figure S4. Interstitial fibrosis in the myocardium of LCWE-injected mice. (A) Masson's trichrome staining of myocardium tissue section from LCWE-injected mice at 12 weeks post-LCWE injection. Scale bars: 500 μm. LV, left ventricle artery; RV, right ventricle.
